# Reorganization of the structural connectome in primary open angle Glaucoma

**DOI:** 10.1016/j.nicl.2020.102419

**Published:** 2020-09-09

**Authors:** Francesco Di Ciò, Francesco Garaci, Silvia Minosse, Luca Passamonti, Alessio Martucci, Simona Lanzafame, Francesca Di Giuliano, Eliseo Picchi, Massimo Cesareo, Maria Giovanna Guerrisi, Roberto Floris, Carlo Nucci, Nicola Toschi

**Affiliations:** a Medical Physics Section, Department of Biomedicine and Prevention, University of Rome “Tor Vergata”, Italy; b Neuroradiology Unit, Department of Biomedicine and Prevention, University of Rome “Tor Vergata”, Rome, Italy; c San Raffaele Cassino, Frosinone, Italy; d Institute of Bioimaging and Molecular Physiology, National Research Council, Milano, Italy; e Department of Clinical Neurosciences, University of Cambridge, Cambridge, UK; f Ophthalmology Unit, Department of Experimental Medicine, University of Rome Tor Vergata, Rome, Italy; g Diagnostic Imaging Unit, Department of Biomedicine and Prevention, University of Rome “Tor Vergata”, Rome, Italy; h Athinoula A. Martinos Center for Biomedical Imaging and Harvard Medical School, Boston, MA, USA

**Keywords:** Structural connectivity, Diffusion MRI, Tractography, Primary open angle glaucoma, Graph theory, Neurodegenerative disease

## Abstract

Primary open angle Glaucoma (POAG) is one of the most common causes of permanent blindness in the world. Recent studies have suggested the hypothesis that POAG is also a central nervous system disorder which may result in additional (i.e., extra-ocular) involvement.

The aim of this study is to assess possible structural, whole-brain connectivity alterations in POAG patients. We evaluated 23 POAG patients and 15 healthy controls by combining multi-shell diffusion weighted imaging, multi-shell, multi-tissue probabilistic tractography, graph theoretical measures and a recently designed ‘disruption index’, which evaluates the global reorganization of brain networks. We also studied the associations between the whole-brain structural connectivity measures and indices of visual acuity including the field index (VFI) and two Optical Coherence Tomography (OCT) parameters, namely the Macula Ganglion Cell Layer (MaculaGCL) and Retinal Nerve Fiber Layer (RNFL) thicknesses.

We found both global and local structural connectivity differences between POAG patients and controls, which extended well beyond the primary visual pathway and were localized in the left calcarine gyrus (clustering coefficient p = 0.036), left lateral occipital cortex (clustering coefficient p = 0.017, local efficiency p = 0.035), right lingual gyrus (clustering coefficient p = 0.009), and right paracentral lobule (clustering coefficient p = 0.009, local efficiency p = 0.018). Group-wise (clustering coefficient, p = 6.59∙10^–7^ and local efficiency p = 6.23·10^-8^) and subject-wise disruption indices (clustering coefficient, p = 0.018 and local efficiency, p = 0.01) also differed between POAG patients and controls. In addition, we found negative associations between RNFL thickness and local measures (clustering coefficient, local efficiency and strength) in the right amygdala (local efficiency p = 0.008, local strength p = 0.016), right inferior temporal gyrus (clustering coefficient p = 0.036, local efficiency p = 0.042), and right temporal pole (local strength p = 0.008).

Overall, we show, in patients with POAG, a whole-brain structural reorganization that spans across a variety of brain regions involved in visual processing, motor control, and emotional/cognitive functions. We also identified a pattern of brain structural changes in relation to POAG clinical severity. Taken together, our findings support the hypothesis that the reduction in visual acuity from POAG can be driven by a combination of local (i.e., in the eye) and more extended (i.e., brain) effects.

## Highlights

• Primary Open Angle Glaucoma is one of the major causes of blindness worldwide.
• Structural connectivity and graph theory allow to analyse brain network changes.
• Disruption index is able to estimate the reorganization of nodal network topology.
• The global network of Primary open angle Glaucoma patients is highly reorganized.
• Primary open angle glaucoma could be considered a neurodegenerative disease.

## 1 Introduction

One of the most common causes of permanent blindness in the world is primary open angle Glaucoma (POAG), an ocular disorder typically characterized by open and normal appearing anterior chamber angle and elevated intraocular pressure (IOP). POAG is a progressive disorder that leads to irreversible loss of optic nerve fibers, retinal ganglion cells, degeneration of the axons in the optic nerve (Nucci et al., 2012) and loss of visual field (Martucci et al., 2018). Several neuroimaging studies have highlighted white-matter (WM) (Boucard et al., 2016, Frezzotti et al., 2014, Giorgio et al., 2018, Haykal et al., 2019) and grey-matter (GM) changes (Chen et al., 2013, Wang et al., 2019) as well as variations in functional brain connectivity (Minosse et al., 2019b, Frezzotti et al., 2016, Giorgio et al., 2020, Wang et al., 2016) in POAG patients relative to healthy controls. Interestingly, the brain areas highlighted in these studies did not exclusively involve the visual pathways but extended to brain regions that have been implicated in complex cognitive and behavioural functions. For Instance, Frezzotti et al (Frezzotti et al., 2014) found higher axial diffusivity (AD) in the middle cerebellar peduncle, corticospinal tract, anterior thalamic radiation and superior longitudinal fascicle in POAG patients relative to controls, and these results were confirmed in additional, subsequent studies (Boucard et al., 2016, Frezzotti et al., 2016, Giorgio et al., 2018). Moreover, Minosse et al (Minosse et al., 2019b), found that POAG patients display whole-brain functional reorganization relative to healthy subjects, and that several graph-theoretical metrics derived from functional networks were able to discriminate well between POAG (Martucci et al., 2018) and controls. Overall, these studies have supported the hypothesis of a brain involvement in POAG which extends beyond the visual pathways to include additional brain networks that are critical for cognition and behaviour.

Diffusion-weighted imaging based tractography is a powerful tool able estimate anatomical connection through long-range white-matter bundles in the human brain. It is also the only non-invasive imaging technique that allows to reconstruct the putative direction of axonal fibers in-vivo, and has provided important insight in a vast number of neurological and neurodegenerative disorders (Cacciola et al., 2019, Nicolas W. Cortes-Penfield et al., 2017, Nigro et al., 2016, Shigemoto et al., 2018). However, the diffusion tensor imaging (DTI) model for estimating the voxel-wise water self-diffusion probability profile (often used in conjunction with either probabilistic or deterministic tractography techniques) relies on the assumption of a one-to-one mapping between each voxel and fiber direction. This assumption is not always adequate when aiming to resolve the high, *meso*-scale structural complexity commonly found within the scale of a voxel. This limitation can be overcome using multi-shell diffusion weighted data acquisition in conjunction with more advanced models such as constrained spherical deconvolution. In particular, the recently presented multi-shell multi-tissue constrained spherical deconvolution (MS-MT-CSD) (Jeurissen et al., 2014) technique has further improved the estimation of voxel wise orientation distribution functions, also incorporating the use of intrinsically generated volume fraction maps for white matter (WM), grey matter (GM) and cerebrospinal fluid (CSF). Once the structural connectome is reconstructed from tractography analysis, a popular strategy to obtain interpretable summary statistics is the subsequent application of graph theoretical analysis (Fornito et al., 2016, Rubinov and Sporns, 2010). This involves the conceptualization of different brain regions as graph nodes, connected by graph edges that represent the streamlines that originated from tractography.

This study aims to evaluate potential, structural whole-brain connectivity changes in POAG. Based on previous imaging studies in POAG, we hypothesized the existence of both local and global structural connectivity changes in POAG (Minosse et al., 2019b, Frezzotti et al., 2016, Wang et al., 2016). Moreover, we expected to find changes in local and global connectivity which extend beyond primary and secondary visual areas (Minosse et al., 2019b, Boucard et al., 2016, Giorgio et al., 2018). In addition, we explored possible associations between local and global graph theoretical metrics and disruption indices with measures of clinical severity such as the visual field index (VFI) and two Optical Coherence Tomography (OCT) derived measures: Macula Ganglion Cell Layer (GCL) and Retinal Nerve Fiber Layer (RNFL) (Martucci et al., 2018) thicknesses.

## 2 Material and methods

### 2.1 Subjects

Twenty-three POAG patients and sixteen healthy controls were enrolled from the Glaucoma Clinic as well as the General Outpatients clinic at the University Hospital “Policlinico Tor Vergata” (Rome, Italy). Subject demographics are described in Table 1. The study protocol was approved by the local Institutional Review Board and adhered to the tenets of the Declaration of Helsinki. All subjects provided written informed consent. After POAG diagnosis, POAG patients were deemed eligible for the current study if they fulfilled the following inclusion criteria: (I) open anterior chamber (Shaffer classification > 20°) (II) transparent ocular media, (III) refractive error <±5 spherical diopters or <±3 cylindrical diopters and (IV) best corrected visual acuity > 0.1 logMAR. Exclusion criteria for POAG patients as well as healthy controls were: (I) hereditary retinal dystrophy, (II) previous or active neurological, cerebrovascular, or neurodegenerative diseases, (III) use of medication that could affect visual field, (IV) pre-proliferative or proliferative diabetic retinopathy, (V) macular degeneration, (VI) retinal vascular diseases, (VII) previous or active optic neuropathies. Normal tension Glaucoma patients were also excluded (Martucci et al., 2018). POAG diagnosis was defined following the European Glaucoma Society criteria European Glaucoma Society Terminology and Guidelines for Glaucoma, 4th Edition - Chapter 2: Classification and terminologySupported by the EGS Foundation, 2017). Patients were treated using topical prostaglandin analogues, carbonic anhydrase inhibitors and beta-blockers, alone or in fixed or unfixed combination.

**Table 1:** Demographic and clinical characteristics of the study population. IOP: intra-ocular pressure (^†^ patients under treatment); POAG (primary open angle Glaucoma). * No group-wise statistical differences in age or sex were found (see Results).

	POAG	Healthy controls
Group size	23	15
Age (years) Mean (range)	62.0 (50 – 72)*	60.2 (50 – 76)*
Sex (male/female)	8 / 15*	9 / 6*
IOP Mean (range)	15.74 (12 – 18)^†^	15.27 (12 – 18)
Disease stage	I (4), II (6), III (6), IV (5), V (2)	–

### 2.2 Ophthalmological examinations

All participants underwent a medical history questionnaire, intraocular pressure (IOP) measurement, best-corrected visual acuity, anterior segment examination, standard automated perimetry tests, ultrasound pachymetry and gonioscopy. Visual Field (VF) evaluation was performed using Humphrey Swedish Interactive Threshold Algorithm (SITA) standard with a 24–2 test point pattern (Carl Zeiss Meditec Inc., Dublin, CA). The visual field index (VFI) is a global metric that estimates the entire VF as a fraction of the normal (maximum) value (100%: normal VF − 0% abolished VF) (Gros-Otero et al., 2015). After pupillary dilation, fundus examination and spectral domain-optical coherence tomography (SD-OCT) using Glaucoma Module Premium Edition (GMPE) software (Heidelberg Retinal Engineering, Dossenheim, Germany) were performed (Martucci et al., 2018), from which we extracted RNFL and GCL thickness values. In detail, GCL values were averaged across nine regions (Fovea, Superior Inner, Nasal Inner, Temporal Inner, Inferior Inner, Superior Outer, Inferior Outer, Temporal Outer, Nasal Outer) and both eyes, and RNFL values were averaged across six regions (nasal, nasal superior, nasal inferior, temporal, temporal superior, temporal inferior) and both eyes (Minosse et al., 2019b) in order to obtain a single, per-subject value for each thickness estimate.

### 2.3 Magnetic Resonance imaging

Magnetic Resonance Imaging (MRI) was performed on 3-Tesla scanner (Achieva 3T Intera, Philips Healthcare, The Netherlands) equipped with 80 mT/m gradients (maximum amplitude), rise time of 200 mT/m/ms and a dedicated 8-channel head coil. The MR protocol included a T1-weighted high-resolution sequence obtained using a three dimensional magnetization prepared rapid acquisition gradient-echo (MPRAGE) sequence with the following parameters: TR = 500 (ms), TE = 50 (ms), flip angle = 8°, FOV = 256 × 240 mm^2^, acquisition and reconstruction voxel size = 1 × 1 × 1.2 mm^3^. Diffusion-weighed imaging was performed using a spin-echo (SE) echo-planar (EPI) single shot sequence with interleaved slice acquisition and the following parameters: FOV = 240x240, matrix = 94x94 voxel, TE = 89 ms, TR = 7774 ms, slice thickness = 2.5 mm, 60 slices, no gap, SENSE reduction factor R = 2. Two distinct b-values (1000 s/mm^2^ and 2500 s/mm^2^) were applied in 64 non coplanar and non collinear directions (32 for each non-zero b-value), for diffusion weighting. In addition, eight non diffusion-weighted reference images (b0 images) were acquired.

### 2.4 MRI data analysis

**Fig. 1:**
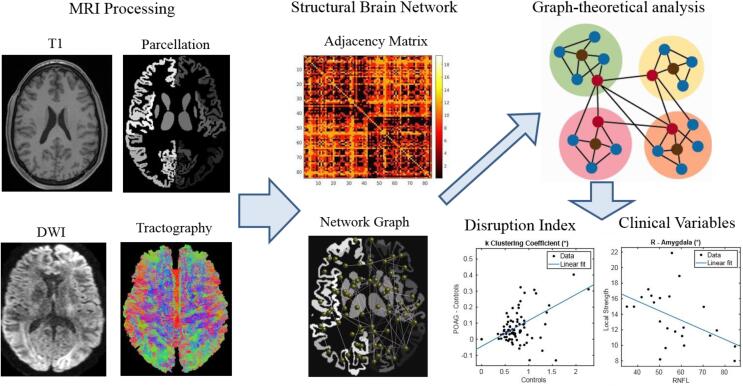
Overall analysis workflow.

The overall analysis workflow is shown in Fig. 1. First, we applied Brain extraction (Smith, 2002) (BET, part of FSL (Jenkinson et al., 2012, Smith et al., 2004)) and segmentation in three tissue types (GM, WM an CSF (Smith et al., 2004, Smith et al., 2012)) employing FAST (also part of FSL) to the T1 weighted image. The T1 weighted images were also passed through the FreeSurfer reconstruction stream (Fischl, 2012) resulting in cortical parcellations based on the Desikan-Killany Atlas. Subcortical parcels were added to the cortical parcellation based on the volumetric segmentation provided by FAST (Patenaude et al., 2011, Smith et al., 2015a, Smith et al., 2004), resulting in a total of 84 parcels. Diffusion-weighted images were corrected for subject motion and eddy-current-induced distortions within the ExploreDTI software (Irfanoglu et al., 2012), including geometric image distortion correction and b-matrix reorientation (Leemans and Jones, 2009) (version 9.5.0 under MATLAB 2018). Successively, the response function (RF) (Jeurissen et al., 2014) was estimated in order to obtain the WM fODF (fiber Orientation Distribution Function) (Jeurissen et al., 2014, Tournier et al., 2004) for multi-shell, multi-tissue CSD. Probabilistic tractography was then performed in MRtrix3 (Tournier et al., 2019) by tracing 100 million of streamlines (Tournier et al., 2010, Smith et al., 2015b, Smith et al., 2012) with the following options: step size 1.25 mm, angle theta 45°, minimum track length 5 mm, maximum track length 250 mm and cut off value of 0.05. The streamlines were then filtered down to 10 million streamlines per subject using Spherical-deconvolution Informed Filtering of Tractograms (SIFT) (Smith et al., 2013). Tractography was performed on a high-performance parallel computing cluster and took approximately 60 days of CPU time.

### 2.5 Connectome analysis

A connectome (i.e. an adjacency matrix, also termed connectivity matrix) was constructed for each subject by mapping the streamline reconstruction onto the 84 regions (Desikan Atlas) (Hagmann et al., 2008). The adjacency matrices were subsequently thresholded at a sparsity value of 10% (Minosse et al., 2019b, Wang et al., 2016), and the following graph-theoretical metrics were calculated for each subject. Local metrics: local strength, betweenness centrality, measures of centrality, local efficiency, clustering coefficient, measures of functional segregation. Global measures: global strength, global clustering coefficient, global efficiency and transitivity (Conti et al., 2019). All metrics were computed using the Brain Connectivity Toolbox (Rubinov and Sporns, 2010).

### 2.6 Disruption index

**Fig. 2:**
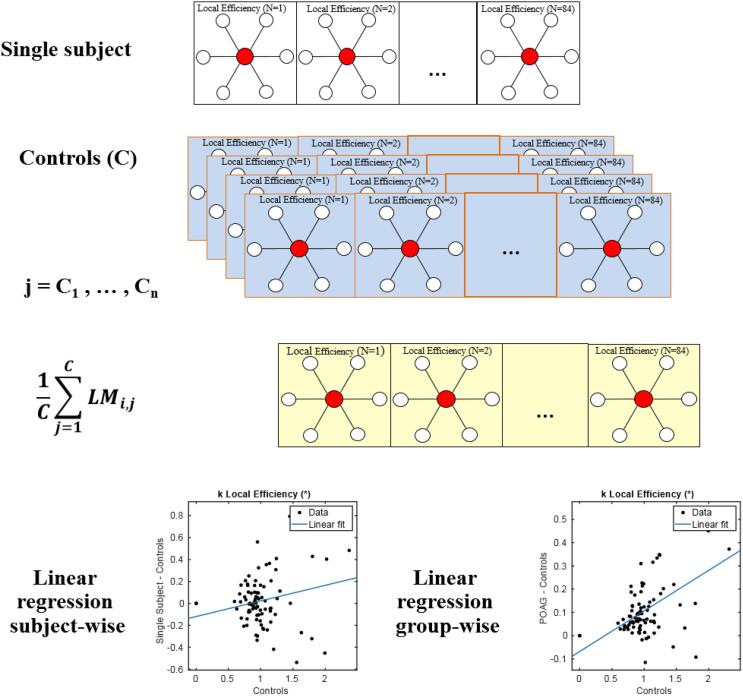
Schematic illustration of computation of the disruption index for one metric. Fourth row: in the linear regression, the independent variable (x-axis) is the mean value (across controls) of a particular graph metric for each region of interest. In case of subject wise regression (left), the dependent variable (y-axis) is the difference, for each region of interest, between the value of a particular graph metric and the mean value (across controls) of a particular graph metric for the same region of interest. In case of group-wise regression, the dependent variable (y-axis) is the difference between the PAOG group mean and the mean of all controls.

The disruption index k estimates the comprehensive reorganization of the nodal network topology of an individual subject compared to the study population. The calculation is illustrated below as well as in Fig. 2. It is computed as the linear regression slope by using single local graph metric values (*LM_i_*) across all nodes as in (Minosse et al., 2019a, Minosse et al., 2019b):

(1) $${\mathrm{LM}}_{i,S}-\frac{1}{C}\sum_{j=1}^{C}{\mathrm{LM}}_{i,j}=k_{i,S}^{0}+k_{i,S}∙\frac{1}{C}\sum_{j=1}^{C}{\mathrm{LM}}_{i,j}+\varepsilon_{i,S}$$

(2) $$\frac{1}{P}\sum_{j=1}^{P}{\mathrm{LM}}_{i,j}-\frac{1}{C}\sum_{j=1}^{C}{\mathrm{LM}}_{i,j}=k_{i}^{0}+k_{i}∙\frac{1}{C}\sum_{j=1}^{C}{\mathrm{LM}}_{i,j}+\varepsilon_{i}$$

where *LM_i,j_* = *C*, *LM_i,j_ = P*, and *LM_i,S_* are the local measures (*i*) for controls (*C*), POAG patients (*P*) and all subjects (*S = C + P*), respectively*. LM_i_* ∈ ℝ^N^, where N is the number of the node (1 ÷ 84). *k_i,S_* and *k_i_* are the disruption indices relative to the graph measures (*i*) for a single subject and for the PAOG patient group, respectively. *k0_i,S_* and *k0_i_* are constant terms and *ε_i,S_* and *ε_i_* are the residual of linear regression. In detail, to obtain an estimate of the global disruption index, the region-wise value for a given local measure is determined. Then, the linear regression slope of the difference between the mean value of that local measure across the PAOG patient group and the corresponding mean value of the control group is computed. Additionally, a subject-wise disruption index can be determined by subtracting the mean control group metric from the metric of the corresponding node of each patient and calculating the linear regression slope of the differences. More details can be found in (Achard et al., 2012, Wang et al., 2016)

### 2.7 Hub analysis

In order to further assess network reorganization, we evaluated the presence or absence of subject-wise hub regions in POAG patients and healthy controls. In order to classify a node as hub, the whole-brain average of each local graph-theoretical metric was computed. Successively, a region was classified as a hub for that particular metric if the average value of that metric value was higher than 1.5 times the whole-brain average.

### 2.8 Statistical analysis.

Local and global graph theoretical metrics as well as disruption indices were compared between the two groups using the non-parametric Mann-Whitney U Test. Effect size was estimated as percent differences between group-wise medians. The presence/absence of the hub in any specific node was compared across groups using Fisher’s exact test. The association between clinical and OCT parameters and local and global metrics as well as disruption indices was assessed using separate linear models for each pair of variables. All regression models included sex and age as nuisance covariates. For regression models, effect size was quantified using Cohen’s *f^2^* measure. All tests which involved multiple local measures were corrected for multiple comparisons across regions using a false discovery rate (FDR) procedure (alpha = 0.05), and p < 0.05 (corrected) was considered statistically significant. In case of global variables, FDR correction was applied across all global metrics and all disruption indices separately, for each OCT variable. Finally, in order to evaluate the ability of graph-theoretical metrics to discriminate between POAG patients and controls, binary logistic regression was used to construct the receiving operating characteristic curve (ROC). Youden’s index was used to estimate the optimal operating point of each ROC curve, which was used to compute sensitivity, specificity, positive predictive value (PPV) and negative predictive value (NPV). All statistical analyses were performed in MATLAB version 9.5.0, (MathWorks, Natick, MA, USA) using scripts developed in-house.

## 3 Results

We found no statistically significant group-wise differences in age (p = 0.42, Mann-Whitney-*U* test) or sex (p = 0.13, Chi-square test).

### 3.1 Global graph theoretical metrics

**Fig. 3:**
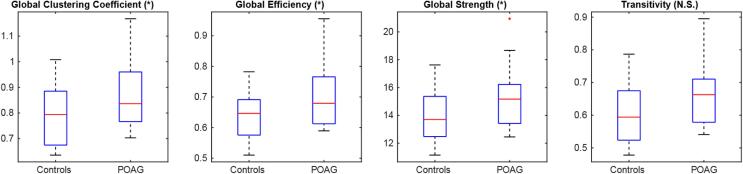
Group-wise global graph-theoretical metrics. (*) p < 0.05 NS: not significant.

In global metrics (Fig. 3), we found statistically significant differences between POAG patients and Controls in the global clustering coefficient (p = 0.042, effect size [POAG > Controls] = 5%,), global efficiency (p = 0.042, effect size [POAG > Controls] = 5%) and global strength (p = 0.045, effect size [POAG > Controls] = 10%). We did not find statistically significant effects in transitivity.

### 3.2 Local graph theoretical metrics

**Fig. 4:**
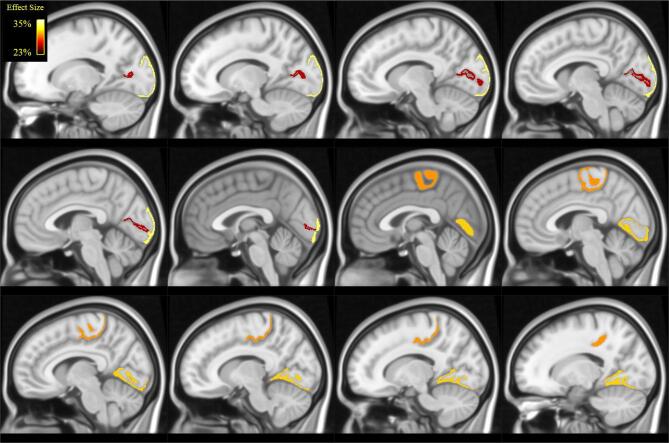
Illustration (in MNI space) of the brain regions in which we found statistically significant differences in in clustering coefficient (see Table 2): lateral occipital cortex, calcarine cortex, lingual gyrus and paracentral. Colour coding reflect effect-size (Table 2).

**Fig. 5:**
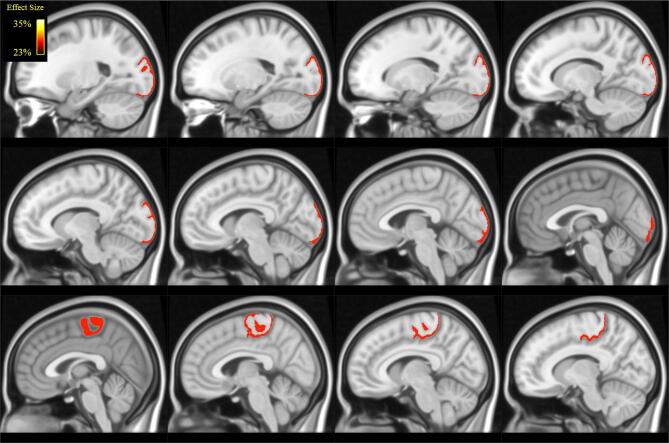
Illustration (in MNI space) of the brain regions in which we found statistically significant differences in in local efficiency (see Table 2): lateral occipital cortex and paracentral lobule. Colour coding reflects effect-size (Table2).

When analysing local metrics (Table 2 and Fig. 4, Fig. 5), we found statistically significant differences between POAG patients and controls for clustering coefficient and local efficiency only. These differences were localized in the left occipital lobe, in the lateral occipital cortex and in the calcarine gyrus. Further differences were localized in the right occipital lobe, in the lingual gyrus and in the right paracentral lobule, whose anterior part is situated within the frontal lobe, whereas the posterior part is located in the parietal lobe.

**Table 2:** Results of Mann-Whitney *U* test across groups in local graph-theoretical measures and related effect sizes (POAG > Controls). N.S. = non-significant.

	Clustering Coefficient		Local Efficiency	
Region	Effect Size	p	Effect Size	p
L -lateral-occipital	33%	0.017	28%	0.035
L - calcarine gyrus	26%	0.036	–	N.S.
R -lingual	31%	0.009	–	N.S.
R -paracentral	30%	0.009	28%	0.018

### 3.3 Disruption indices

**Fig. 6:**
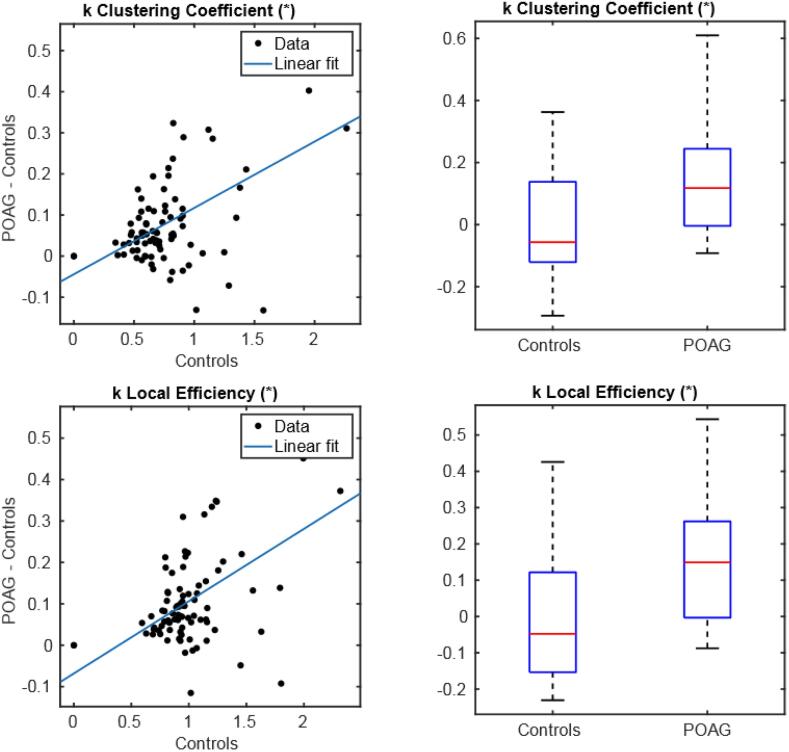
Group-wise disruption index (left) and group-wise differences (right) in subject-wise disruption index between controls and POAG patients (right). (*) p-value < 0.05, (**) p-value < 0.01.

Group-wise disruption indices were seen to be statistically different from 0 for both clustering coefficient (p = 6.59∙10^–7^, k = 0,16) and local efficiency (p = 6.23 10^-8^, k = 0,18) (with a positive regression slope) indicating global network reorganization in POAG patients as compared to controls. Similarly, subject-wise disruption indices presented a statistically significant group-wise difference for clustering coefficient (p = 0.018, effect size [POAG > Controls] = 148%) and Local Efficiency (p = 0.01, effect size [POAG > Controls] = 132%) (Fig. 6).

### 3.4 Hub analysis

**Fig. 7:**
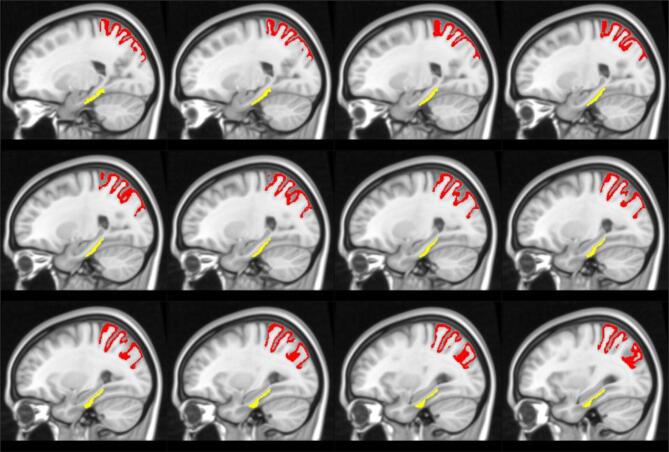
Illustration (in MNI space) of the brain regions that emerged as a hub healthy control but not in POAG patients (in yellow) and in POAG patients but not in health controls (in red). (For interpretation of the references to colour in this figure legend, the reader is referred to the web version of this article.)

Hub analysis resulted in the presence of one single hub per group, in different regions. The right parahippocampal gyrus (p = 0.043) of the temporal lobe emerged as local Efficiency hub in the control, but not in the POAG group, while the right superior parietal lobule (p = 0.039) of the parietal lobe emerged as betweenness centrality hub in the POAG, but not in the control group (Fig. 7).

### 3.5 Association of brain measures with clinical and OCT variables

**Fig. 8:**
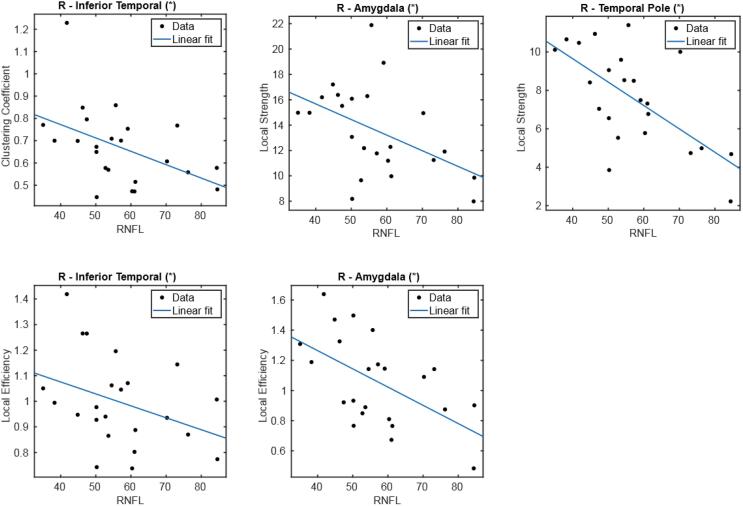
Linear regression representing the negative association between local metrics (clustering coefficient, local efficiency and local strength) and RNFL. (*) p-value < 0.05.

We found no statistically significant association between clinical variables and global metrics or between clinical variables and disruption index. However, we found a statistically significant, negative association between several local metrics and RNFL (Fig. 8) thickness in three regions (Table 3): amygdala (local efficiency p = 0.008, local strength p = 0.016), inferior temporal cortex (clustering coefficient p = 0.036, local efficiency p = 0.042) and temporal pole (local strength p = 0.008). All these regions are located in the right temporal lobe.

**Table 3:** Results of linear regression of local graph theoretical measures against RNFL thickness. All associations were negative.

Region	Measure	*Cohen’s f^2^*	*p*
R-Amygdala	Local Efficiency	1.519	0.008
	Local Strength	1.274	0.016
R-Inferior temporal	Local Clustering Coefficient	1.263	0.037
	Local Efficiency	1.360	0.042
R-Temporal pole	Local Strength	1.304	0.008

### 3.6 ROC analysis

**Fig. 9:**
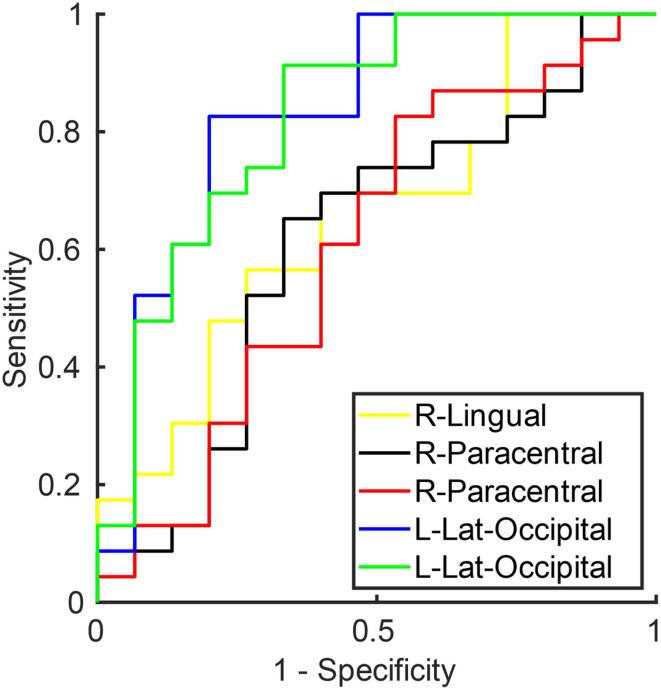
ROC curve generating when using the top 5 performing (in terms of AUC) local measures (Table 5) in the differentiation task between POAG and Controls.

ROC analysis of global metrics and disruption indices (Table 4) yielded a medium discriminative power as determined by the Area under the ROC curve (maximum AUC = 0.75, for the disruption index calculated from local efficiency). Local graph theoretical measures performed best in discriminating between POAG patients and controls, with AUC values as high as 0.86 (see Table 5 for the top 10 AUC values and Fig. 9 for a graphical depiction of the corresponding ROC curves, top 5 only).

**Table 4:** Results of ROC analysis for global graph theoretical measures and disruption indices. AUC = area under the ROC curve; PPV = positive predictive value; NPV = negative predictive value. AUC values are ordered from high to low, top-down.

Measure	AUC	Accuracy	Sensitivity	Specificity	PPV	NPV
k Local Efficiency	0.751	0.684	0.652	0.733	0.789	0.579
k Clustering coefficient	0.730	0.763	0.913	0.533	0.750	0.800
Global Clustering coefficient	0.699	0.658	0.609	0.733	0.778	0.550
Global Efficiency	0.699	0.684	0.696	0.667	0.762	0.588
Global Strength	0.696	0.684	0.696	0.667	0.762	0.588
Transitivity	0.664	0.605	0.565	0.667	0.722	0.500
k Local Strength	0.600	0.658	0.783	0.467	0.692	0.583
k betweenness centrality	0.458	0.526	0.435	0.667	0.667	0.435

**Table 5:** Results of ROC analysis for local graph theoretical measures (Top 15 AUC values). AUC = area under the curve; PPV = positive predictive value; NPV = negative predictive value. AUC values are ordered from high to low, top-down.

Region	Measure	AUC	Accuracy	Sensitivity	Specificity	PPV	NPV
R lingual	Local Clustering Coefficient	0.864	0.816	0.783	0.867	0.900	0.722
R paracentral	Local Clustering Coefficient	0.861	0.868	0.870	0.867	0.909	0.813
R paracentral	Local Efficiency	0.861	0.842	0.870	0.800	0.870	0.800
L lateral-occipital	Local Clustering Coefficient	0.835	0.816	0.826	0.800	0.864	0.750
L lateral-occipital	Local Efficiency	0.826	0.816	0.913	0.667	0.808	0.833
L lingual	Local Strength	0.820	0.816	0.826	0.800	0.864	0.750
L calcarine gyrus	Local Clustering Coefficient	0.806	0.763	0.739	0.800	0.850	0.667
R lingual	Local Efficiency	0.788	0.789	0.739	0.867	0.895	0.684
L Cerebellum-Cortex	Local Strength	0.788	0.789	0.870	0.667	0.800	0.769
R Cerebellum Cortex	Local Strength	0.786	0.737	0.739	0.733	0.810	0.647
L calcarine gyrus	Local Efficiency	0.780	0.711	0.652	0.800	0.833	0.600
L inferior temporal	Local Strength	0.771	0.763	0.739	0.800	0.850	0.667
L inferior temporal	Local Efficiency	0.768	0.737	0.739	0.733	0.810	0.647
R superior parietal	Local Strength	0.768	0.763	0.826	0.667	0.792	0.714
R Caudate	Local Clustering Coefficient	0.759	0.763	0.783	0.733	0.818	0.688

## 4 Discussion

In this study, we assessed the whole-brain structural network deficits across the spectrum of severity in POAG by combining: 1) diffusion based tractography using a multi-shell, multi-tissue constrained spherical deconvolution model, 2) graph-theory analyses and 3) a recently defined whole brain hub disruption index. Brain reorganization was also assessed in terms of presence/absence of hubs in specific brain regions. Additionally, we evaluated the associations among clinical parameters and graph theoretical measures as well as disruption indices. Moreover, we investigated the ability of measures derived from structural connectivity analysis to discriminate between POAG patients and controls. Overall, we found a reorganization of structural brain networks in POAG which reaches well beyond the visual pathways, corroborating the hypothesis of a brain-wide involvement in POAG. This is supported e.g. by differences between healthy controls and POAG patients in global graph network measures as well as differences in disruption indices. Interestingly, as compared to functional connectivity studies (Minosse et al., 2019b, Wang et al., 2016), we found higher disruption indices in POAG as compared to controls, highlighting a possible complementary role and significance of functional vs. structural connectivity in the analysis of subtle brain changes such as the ones which may be underlying brain involvement in POAG.

The calcarine gyrus is situated in the medial part of the occipital lobe, and corresponds to the primary visual cortex (V1, Brodmann area 17). It receives afferent fibers from the lateral geniculate nucleus, the most important “station” where the optic tracts arrive. It is a fundamental component of the visual pathway as well as of vision-related function. The ventral and dorsal streams of V1 originate from the IVα and IVβ layers, respectively. In addition, V1 also sends afference to other visual association areas (Kandel, 2013a). Therefore, it could be speculated that a degeneration of the visual pathway (Boucard et al., 2016, Giorgio et al., 2018), could lead this region to increase its functional segregation ability in a compensatory capacity. In support of this hypothesis, a previous study highlighted that, before developing atrophy, a temporary compensatory hypertrophy is observed in several brain structures of early POAG patients (Williams et al., 2013).

We also found group-wise differences in clustering coefficient in the lingual gyrus. The anterior part of this region plays an important role in topographical recognition, i.e. the ability to orient oneself the surroundings, as evidenced by several lesion studies (Mendez and Cherrier, 2003, Takahashi and Kawamura, 2002), showing topographical disorientation. Evidence for changes in the lingual gyrus in POAG patients has been previously shown in morphometry as well as functional studies (Chen et al., 2013, Jiang et al., 2017, Zhou et al., 2016). Along with the observation of orientation difficulties in POAG patients (Friedman et al., 2007, Ramulu, 2009, Sotimehin and Ramulu, 2018), we speculate that the higher clustering coefficient in this region might be related to the absence of the local efficiency hub in the parahippocampal gyrus, which is present in healthy controls but not in POAG patients. The parahippocampal place area, part of the parahippocampal gyrus, plays a fundamental role in the perception of local visual environment (Epstein and Kanwisher, 1998, Mégevand et al., 2014), and there are reports of topographical disorientation after suffering a parahippocampal lesion (Barrash, 1998, Ishii et al., 2017, Luzzi et al., 2000). Therefore, we speculate that the modification in this hub might act as a modifier in the functional segregation of the lingual gyrus. Moreover, several studies focused on Alzheimer’s Disease have highlighted a reduction and thinning of the hippocampal gyrus (Krumm et al., 2016, McLachlan et al., 2018, Thangavel et al., 2008). This further supports the idea that POAG could be part of a heterogeneous group of disconnection syndromes which has recently been hypothesized to include a range of dementias (Minosse et al., 2019b).

We also found a betweenness centrality hub in the superior parietal gyrus is in POAG patients only. The superior parietal gyrus is intercalated in the dorsal pathway. This area is supposed to be involved in visual motion, spatial processing and visual attention (Kandel, 2013b, Lester and Dassonville, 2014). Furthermore, it also provides visual information to the motor system (Kandel, 2013b), and changes in this region were shown in other POAG studies focused on morphometry or functional measures (Chen et al., 2019, Chen et al., 2013, Jiang et al., 2017, Wang et al., 2019). Additionally, due to its role in spatial processing, this region plays important role in topographical recognition. Its presence as hub in POAG patients further supports the hypothesis of a brain substrate underlying dysfunctions experienced by POAG patients.

In POAG, we also observed a higher clustering coefficient and local efficiency in the lateral occipital cortex. This region is located in the visual association area (V2, Brodmann area 18), along with the lingual gyrus. V2 is supposed to play an important role in the integration of visual information and generation of conscious perceptions, and previous studies have highlighted its role in POAG (Giorgio et al., 2018, Jiang et al., 2017, Zhou et al., 2016). The lateral occipital cortex not only plays a fundamental role in object recognition (Grill-Spector et al., 2001), but also in face recognition (Nagy et al., 2012). Interestingly, patients with POAG have an impairment in face recognition ability (Minosse et al., 2019b, Glen et al., 2012), again lending further support of a possible a brain substrate for this impairment in POAG. This finding is also connected with the negative association we observed between local graph-theoretical measures in the right inferior temporal cortex and RNFL thickness. This region is a key player in object recognition, and it is part of ventral pathways that begin in V1 (calcarine cortex), pass through V2 (lateral occipital cortex, lingual gyrus and fusiform gyrus) and the temporal occipital cortex, then reaching the inferior temporal cortex. Furthermore, the inferior temporal cortex also has connection with the parahippocampal gyrus and the perirhinal cortex, through which it connects with the hippocampus. Moreover, the inferior temporal cortex is connected directly and indirectly (through to the perirhinal cortex) to the amygdala (Kandel, 2013c). In this context, primate studies have shown that the inferior cortex plays an important role in face recognition (Desimone et al., 1984, Rolls et al., 1994). Additionally, it has been shown that lesions in this area led to prosopagnosia (Purves et al., 2001), hence supporting a similar role in humans. Therefore, the higher clustering coefficient and local efficiency as a function of lower RNFL thickness in POAG (Li et al., 2014, Williams et al., 2013), point toward a link between POAG and changes in the lateral occipital area which may concur to impaired face recognition.

It is also important to note that local graph theoretical measures in the right amygdala showed a negative correlation with RNFL thickness. The amygdala is involved in several functions such as such as emotion and behaviour. It is also believed to have a role in processing facial emotions (Liu et al., 2015, Wu et al., 2016). This is in accordance with existing evidence for difficulties in recognizing facial emotions in POAG patients (Schafer et al., 2018). Interestingly, it has also been observed that POAG patients have higher probability to suffer from depression (Cesareo et al., 2015, Thau et al., 2018) and anxiety (Zhang et al., 2017). This is commonly explained uniquely as a psychological and clinical consequence of vision impairment. Still, it is believed that the temporal pole (which along with the amygdala is part of the limbic system (Olson et al., 2007)) plays a role in face recognition and encoding (Olson et al., 2007, Von Der Heide et al., 2013). While the present study only allows to infer associations as opposed to causal relationships, these observations may offer alternative explanations for the difficulties of POAG patients in face recognition as well as for the pathogenesis of depressive and anxiety episodes.

Also, the paracentral lobule is located in the posterior part of the frontal lobe and in the anterior part of the parietal lobe. Its anterior part is located in the primary motor cortex (Spasojević et al., 2013) (Brodmann area 4) and is involved in voluntary movements. In particular, this region plays a role in voluntary movements of contralateral leg and foot. In this context, it has been shown that POAG patients experience difficulties in visuomotor coordination (Trivedi et al., 2019), increasing the risk of falling. The differences in local graph-theoretical measures we found in the paracentral lobe, consistent with previous papers which employed different imaging techniques (Chen et al., 2017, Chen et al., 2013, Song et al., 2018, Wang et al., 2016), lend further support for the hypothesis of a brain substrate underlying the multiple disabilities experienced by Glaucoma patients (Nucci et al., 2015, Raffaele et al., 2018, Sotimehin and Ramulu, 2018). Finally, while in our findings the regions which displayed negative association with RNFL appear to be located in the right hemisphere, inconsideration of the small sample size this possible lateralization should be considered a possible trend to be confirmed in a larger population.

In this context, our study is affected by some limitations. It included a relatively small number of subjects and should therefore be considered exploratory – to better assess the impact of our finding, a larger sample size and a longitudinal design would be required. The latter would also aid in detecting causal / mechanistic explanations for the associations between POAG and brain changes shown in our as well as other studies. As opposed to DTI, CSD poses higher demands on scan and computing time, and it is well known that, in general, tractography cannot reconstruct axons directly, but rather indirectly estimates fiber trajectories from measured water self-diffusion profiles. In this respect, multi-shell, multi-tissue CSD offers state-of-the-art precision and has the advantage of being a mainly data-driven method. Furthermore, to the best of our knowledge, our study is the first to employ the disruption index idea in conjunction with structural connectivity in POAG patients. Therefore, this finding needs further investigation in order to support its robustness.

Taken together, the statistically significant differences found in this study highlight complex modification of the structural connectivity network in Glaucoma. While our findings point to the disease itself as a modifier of these network properties, a univocal interpretation of their (possibly causal) implication within the pathogenic process remains elusive. In particular, it could be equally speculated that their underlying biological substrate may be compensatory, the by-product of complex homeostatic mechanisms, or the by-product of a run-away network in the presence of disease. In this context, the modifications we observed share some similarities with what is observed in e.g. Alzheimer’s Disease (Cope et al., 2018, Crossley et al., 2014). However, a common pattern of connectivity alterations across neurodegenerative disease has yet to be discovered (Stam, 2014). Also, it has been highlighted (Cope et al., 2018) that hub regions might be prominently involved in neurodegenerative disease. From a biological perspective, hubs are more “expensive” in term of metabolism and blood flow (Alexander-Bloch et al., 2013, Van Den Heuvel et al., 2012, van den Heuvel and Sporns, 2011) be therefore more vulnerable to disease processes. At the same time such regions often have a more prominent role in cognitive tasks and adaptive behaviour, and their deterioration may therefore support a large share of symptomatology experienced by POAG patients.

## 5 Conclusion

In conclusion, our structural connectivity findings lend strong further support to the hypothesis of a brain-wide involvement in POAG which reaches well beyond the visual system. While no direct causality can be inferred from our cross-sectional study, our findings could support the additional interpretation of POAG as neurodegenerative disease, to be confirmed and further characterized in longitudinal designs.

## Funding

LP is supported by the 10.13039/501100007155Medical Research Council (MRC), UK (MR/P01271X/1).

## CRediT authorship contribution statement

**Francesco Di Ciò:** Formal analysis, Investigation, Writing - original draft, Writing - review & editing. **Francesco Garaci:** Conceptualization, Methodology, Writing - review & editing, Supervision. **Silvia Minosse:** Methodology, Writing - review & editing. **Luca Passamonti:** Writing - review & editing. **Alessio Martucci:** Investigation, Writing - review & editing. **Simona Lanzafame:** Investigation. **Francesca Di Giuliano:** Investigation, Writing - review & editing. **Eliseo Picchi:** Investigation, Writing - review & editing. **Massimo Cesareo:** Investigation, Writing - review & editing. **Maria Giovanna Guerrisi:** Writing - review & editing. **Roberto Floris:** Writing - review & editing, Supervision. **Carlo Nucci:** Conceptualization, Investigation, Methodology, Writing - review & editing, Supervision. **Nicola Toschi:** Conceptualization, Methodology, Formal analysis, Writing - original draft, Writing - review & editing, Supervision.

## Declaration of Competing Interest

The authors declare that they have no known competing financial interests or personal relationships that could have appeared to influence the work reported in this paper.
